# The antidepressant effects and serum metabonomics of bifid triple viable capsule in a rat model of chronic unpredictable mild stress

**DOI:** 10.3389/fnut.2022.947697

**Published:** 2022-09-15

**Authors:** Qinpeng Bu, Jingkai Zhang, Xiang Guo, Yifei Feng, Huan Yan, Weimin Cheng, Zhitao Feng, Meiqun Cao

**Affiliations:** ^1^Third-Grade Pharmacological Laboratory on Chinese Medicine Approved by State Administration of Traditional Chinese Medicine, Medical College of China Three Gorges University, Yichang, Hubei, China; ^2^Graduate School of Guangxi University of Chinese Medicine, Nanning, Guangxi, China; ^3^Shenzhen Institute of Geriatrics, The First Affiliated Hospital of Shenzhen University, Shenzhen, Guangdong, China; ^4^Department of Hematology, The First Affiliated Hospital of Guangxi University of Traditional Chinese Medicine, Nanning, Guangxi, China

**Keywords:** depression, bifid triple viable capsule, hippocampal damage, serum metabolomics, chronic unpredictable mild stress

## Abstract

**Background:**

Probiotics have shown potential antidepressant effects. This study evaluated the effect and probable mechanisms of bifid triple viable capsules (BTVCs) on a rat model of chronic unpredictable mild stress (CUMS).

**Materials and methods:**

Rats were randomly divided into Normal, CUMS model, fluoxetine hydrochloride (FLX), BTVCs, and FLX+BTVCs groups. Depressive-like behaviours, pathological changes in the hippocampus, changes in serum metabolites and potential biomarkers, and metabolic pathways were detected *via* behavioural tests, haematoxylin-eosin staining, nissl staining, non-targetted metabolomics, and ingenuity pathway analysis (IPA).

**Results:**

The rats displayed depressive-like behaviours after CUMS exposure, but BTVCs ameliorated the depressive-like behaviours. In addition, the pathological results showed that the hippocampal tissue was damaged in rats after CUMS exposure and that the damage was effectively alleviated by treatment with BTVCs. A total of 20 potential biomarkers were identified. Treatment with BTVCs regulated D-phenylalanine, methoxyeugenol, (±)-myristoylcarnitine, 18:3 (6Z, 9Z, 12Z) /P-18:1 (11Z), propionyl-L-carnitine, and arachidonic acid (AA) concentrations, all compounds that are involved with biosynthesis of unsaturated fatty acids, glycerophospholipid metabolism, linoleic acid metabolism and AA metabolism. The IPA demonstrated that endothelin-1 signalling and cyclic adenosine monophosphate response element binding protein (CREB) signalling in neurons may be involved in the development of depression.

**Conclusion:**

Our findings suggest that BTVCs can alleviate depressive-like behaviours, restore damage to the hippocampus in CUMS rats and regulate serum metabolism, which may be related to endothelin-1 signalling or CREB signalling in neurons.

## Introduction

Depression is a frequently recurrent psychiatric disorder characterised by feelings of pessimism, despair, anhedonia, and even suicidal ideation (1). The 12-month prevalence of major depressive disorder varies considerably across countries but is approximately 6% worldwide. The lifetime risk of depression is 15- -18%, and one in ten patients, on average, presents with depressive symptoms (2). According to the World Health Organization, unipolar depressive disorders ranked as the third leading cause of the global disease burden in 2004 and predicted as the leading cause by 2030 (3). Selective serotonin reuptake inhibitors (SSRIs) are still the mainstay of medical management, but side effects and the risk of adverse drug reactions have drawn concerns (4).

Probiotics can alleviate depressive-like behaviours (5, 6) and avoid side effects and addictions, in contrast to current treatments (7). Probiotics have been proven to attenuate depressive-like behaviour by modulating imbalances in gut microbiota (8). In recent years, preclinical studies have shown that probiotics can effectively treat neurological diseases. Wallace et al. demonstrated that probiotics could alleviate symptoms of depression (9). The efficacies of probiotics and prebiotics on depression have been validated in various preclinical studies and clinical trials (10). Bifidobacterium, a probiotic, plays a fundamental role in maintaining the gut microbiota ecosystem in humans and animals, as it alleviates various diseases by changing the composition of intestinal microflora (11). Zhu et al. reported that Bifidobacterium has beneficial effects on cognition *via* the concentration increase of brain-derived neurotrophic factor (BDNF) and modulation of the gut microbiome (12). A placebo-controlled trial showed that probiotic *Bifidobacterium longum* NCC3001 reduced depression scores and responses to negative emotional stimuli in multiple brain areas, including the amygdala and fronto-limbic regions. However, to date, little evidence has been found associating the therapeutic effect of Bifidobacterium on depression at the level of serum small-molecule metabolites.

Metabolomics, a branch of omics science that systematically analyses the concentration profiles of small-molecule endogenous metabolites generated by living systems, is a promising approach for identifying new biomarkers and novel metabolic pathways in several diseases (13, 14). In the past decade, the rapid development of liquid chromatography–mass spectrometry (LC–MS) has facilitated non-targetted metabolomics. By mining metabolomes more deeply, researchers are now primed to uncover key metabolites and their associations with diseases (15, 16). Metabolomics now has unique and established advantages for developing biomarkers for several diseases, and investigating the association between phenotype and metabolomics changes (17, 18). Serum metabolomics has been widely used in diagnostic and treatment studies on depression (19). Metabolomics may be a valuable tool for predicting antidepressant outcomes (20). However, few studies have investigated the association between Bifidobacterium and serum metabolites in depression.

Bifid triple viable capsule (BTVC) is a kind of probiotics composed of bifidobacterium, Lactobacillus and enterococcus. In this study, LC–MS serum metabolomics was used to explore the therapeutic effect of BTVC on chronic unpredictable mild stress (CUMS) rats, and to reveal the potential mechanism of BTVCs in the treatment of depression from the metabolite level.

## Materials and methods

### Animals

Adult male Sprague–Dawley (SD) rats (200∼300 g) were purchased from the Experimental Animal Center of China Three Gorges University. Licences no. SCXK (E) 2017-0061. The experimental protocol was approved by the Ethical Committee in Research Medical College of China Three Gorges University of Medical Sciences (NO.202012B0A).

### Chemicals and reagents

A concentration of 0.2 mg/ml fluoxetine hydrochloride (FLX, Eli Lilly Pharmaceutical Co., Ltd, Suzhou) in deionised water was (21). Bifid triple viable capsules (BTVCs, Shangyao Xinyi Pharmaceutical Co., Ltd, Shanghai) were prepared in a 2 mg/ml suspension in deionised water. Mass spectrometry grade methanol (Fisher Scientific, USA), mass spectrometry acetonitrile (Fisher Scientific, USA), and formic acid (Sigma, USA) were used.

### Equipment

A Triple TOF^®^ 6600 high-resolution mass spectrometry system (AB SCIEX, USA) equipped with an ACQUITY UPLC I-Class ultrahigh-performance liquid system (Waters, USA) and Analyst^®^ TF data acquisition software (AB SCIEX, USA) was used. We used a 5417R centrifuge (Eppendorf, Germany); Vortex Mixer T1 vortex oscillator (Titan SCIENTIFIC LAB); TIMI-10K micromini centrifuge (Titan SCIENTIFIC LAB); and a Labconco centrifugal concentrator (Labconco, USA). Ultrapure water (18.2 MΩ•cm) was prepared using a Milli-Q purified water system (Merck Millipore, USA).

### Drug treatment and groups

**FIGURE 1:**
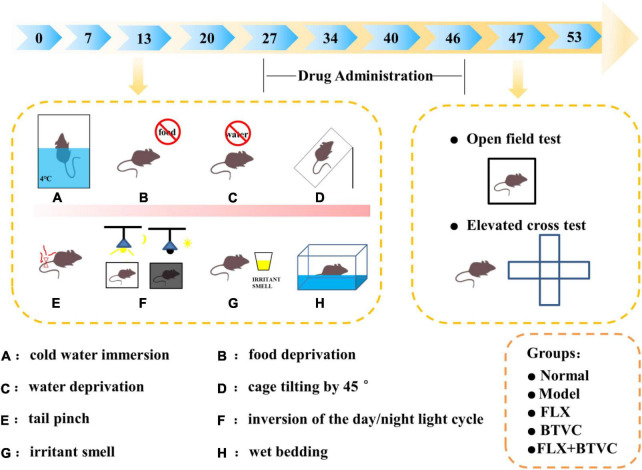
The experimental timeline. **(A–H)** The CUMS procedure.

The FLX solution was given to the animals by oral gavage. Drugs were prepared as specified for rat weight. Rats were randomly assigned to one of the following groups: Normal group, CUMS model group, FLX group (2 mg/kg/d, FLX), BTVCs group (20 mg/kg/d, BTVC), FLX+BTVCs group (2 mg/kg/d FLX + 20 mg/kg/d BTVCs). Each group contained 5 rats. All rats were given agents by gavage at a dose-dependent on body weight once daily for 20 days. Rats in the control and model groups were fed deionised water. Rats were kept under standardised temperature (23 ± 1°C) and a 12 h light/dark cycle (lights on 07:00 –19:00) and had free access to food. The rats lived in abnormally hygienic “specific pathogen free” (SPF) facilities. The experimental timeline is shown in Figure 1.

### Chronic unpredictable mild stress procedure

Except for Normal rats, all groups underwent daily exposures in random order to one of the following chronic unpredictable mild stress for 34 days: 24-h fasting, 24-h water-deprivation, 5-min ice water swimming at 4°C, 2-min tail clamping (1 cm from the tail root), 24 h of a reversed light/dark cycle, 24 h of the cage being tilted, 24-h strange smell (glacial acetic acid sprayed on the litter for 24 h) and 24-h damp bedding. CUMS was randomly performed as summarised in Figure 1 described the experimental schedule (22–24).

### Behavioural tests

The rats were weighed weekly. After 46 days of CUMS exposure, the neurological behaviours of rats were evaluated in the open field test (OFT) and elevated plus-maze (EPM) test. The OFT and EPM tests were recorded, results analysed with Top Scanlife.

The OFT was used to measure exploratory behaviour and general behaviours. Three days before the start of the experiment, the rats were placed in the experimental environment for 5 min every day. The apparatus was a 40 cm × 40 cm × 47 cm box with normal lighting and temperature. A video recording system was stationed above the apparatus to capture the movement of rats within the box. Subsequently, each rat was placed in the centre of the open field, and its movements were video-recorded for 5 min. The apparatus was cleaned between subjects to remove the odour of the previously tested rat. During the test, the observers stayed away from the apparatus. Upon-the OFT finished, the video was analysed using Top Scanlife, and the distance travelled in the centre area, total distance travelled and latency to move were assessed.

The EPM was elevated 50 cm above the ground, constructed from wood, and consisted of two open arms (50 cm × 15 cm) and two enclosed arms (50 cm × 15 cm × 40 cm) with an open top. Rats were placed in the centre of the apparatus, facing one of the open arms, and allowed 5 min of free exploration. Their activity was observed and recorded for 5 min. The percentage of time spent in the open arms was calculated as the percentage of the total time the rat spent on the maze [time in the open arms/ (time in open arms+time in the closed arms) × 100], and the percentage of open arm entries were calculated as a percentage of the total number of rat entries into the maze [number of open arm entries/ (number of open arm entries+number of closed arm entries) × 100] (25).

### Sample collection

After the last behavioural assessment, the blood was collected by the abdominal aortic method. Serum was separated by centrifugation (3,500 rpm, 10 min), and the supernatant was stored at −80°C until analysis. Each brain was dissected on ice; the left hemisphere was removed and fixed with 4% paraformaldehyde solution until tissue sections were obtained. Right hippocampal tissue was isolated and stored at −80°C.

### Paraffin sectioning

The left hemispheres of the brains of the experimental animals were placed in 4% paraformaldehyde, dehydrated with a gradient of alcohol and xylene, and then embedded in paraffin. After the wax solidified, the wax block was placed on a paraffin microtome to slice; slices were baked in a 60°C oven.

### Hematoxylin and eosin staining

The slices were washed 2 times with xylene (20 min per wash) and anhydrous ethanol (10 min per wash), and then the slices were immersed in 95% alcohol for 5 min, 90% alcohol for 5 min, 80% alcohol for 5 min, 70% alcohol for 5 min, and distilled water. The slices were stained in Harris haematoxylin for 3–8 min, washed with tap water, washed 1% hydrochloric acid alcohol for a few seconds, washed with tap water, stained blue with 0.6% ammonia water, and washed again with tap water. Then, the slices were stained in eosin staining solution for 1–3 min, placed in 95% alcohol (5 min per time), anhydrous ethanol (5 min per time), and twice in xylene (5 min per time) for dehydration and to render transparent, then taken out of the xylene and naturally dried. The slices were sealed with neutral resin.

### Nissl stain

The slices were washed in xylene (20 min per wash) and washed twice in anhydrous ethanol (5 min per wash), placed in 75% alcohol for 5 min, and washed with running water. The tissue slices were put into toluidine blue for 5 min, washed with water, and differentiated with 1% glacial acetic acid. The reaction was terminated by washing with tap water. After washing with tap water, the slices were dried in an oven. The slices were immersed in xylene for 5 min and sealed with neutral resin. Elemental composition was calculated from the high-resolution spectra using CasaXPS with measurements.

### Metabolomics

Metabolites were extracted from serum samples. Briefly, cold methanol containing internal standards: deuterated carnitine, FFA, CDCA, CA, Trp, Phe, LPC 19:0, SM 12:0, and choline was added to 50 μL serum. The samples were centrifuged at 14,000 × *g* for 15 min at 4°C, and then the supernatant was subjected to LC-MS. For non-targetted metabolomics assays, an ultra-performance liquid chromatography (UPLC, Waters, USA) system coupled with a Q-TOF high-resolution mass spectrometer (Triple TOF 6600, AB SCIEX, USA) was employed. A more detailed description can be found in the Supplementary material. For quality control, an equal volume of all the serum samples was mixed and inserted per 10 samples in the analytical process to evaluate the reliability of the whole procedure including sample preparation and UPLC-HRMS analysis.

### Ingenuity pathway analysis

The canonical pathways enriched by the differential metabolites were detected with the Ingenuity Pathway Analysis (IPA) suite (version 1.0, QIAGEN, USA). IPA is a web-based software application^1^ that identifies biological pathways and functions relevant to biomolecules of interest. The PubChem CID, *p*-value and fold change (FC) of each metabolite were uploaded to the database to construct a core analysis, and then a list of differential metabolites was uploaded to the IPA along with their Human Metabolome Database (HMDB) identification, FDR (false discovery rate) and logarithmic fold change. Enriched pathways of differential metabolites was generated based on the Ingenuity Pathway Knowledge Database, elucidating the potential targets and pathways of BTVCs in the treatment of depression.

### Statistical analysis

Statistical analysis was performed using SPSS 19.0 software (Chicago, IL, USA). Data were expressed as the mean ± standard deviation ($\bar{\text{x}}\pm$ SD). If the data were normally distributed, comparisons were performed between two groups using the independent-sample *t*-test and among three or more groups using the one-way analyses of variance (ANOVA) test. Pairwise comparisons were made using the least significant difference (LSD) *post-hoc* test after one-way ANOVA. If the data were not normally distributed, the non-parametric Kruskal-Wallis test was used to analyse data. The statistical significance was set at *P* < 0.05.

## Results

### Bifid triple viable capsule improved the behavioural changes of chronic unpredictable mild stress rats

**FIGURE 2:**
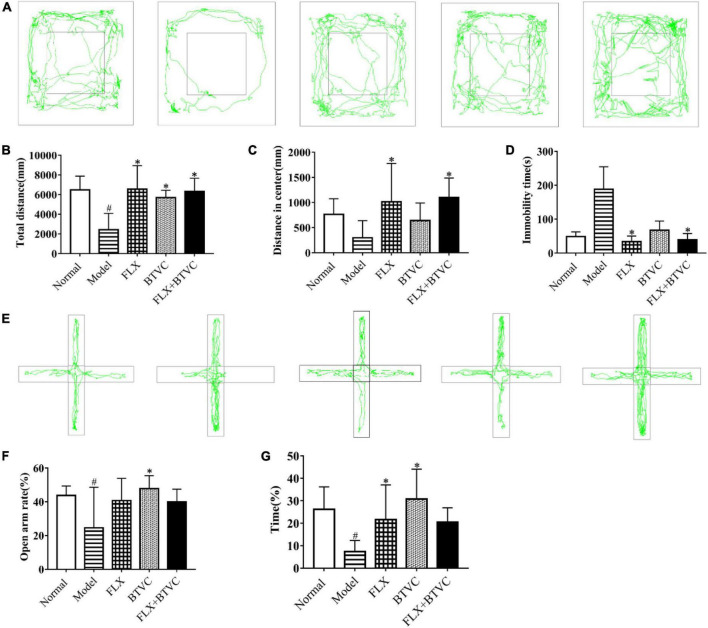
Behavioural test, **(A–D)** the open field test, **(E–G)** the elevated plus-maze test. Representative figure of rats’ tracks **(A)**, the total distance travelled **(B)**, the distance travelled in the central area **(C)**, immobility time in the open field test **(D)**. The movement trajectory of each groups in the elevated plus-maze, the vertical arms are closed arms, the horizontal arms are open arms, and the middle is the central area **(E)**, the ratio of the number of times the rats in each group enter the open arm in the elevated plus-maze **(F)**, the time ratio of staying in the open arm in the elevated plus-maze **(G)**. Compared with the Normal group, ^#^*P* < 0.05; compared with the Model group,*P < 0.05.

As shown in Figure 2A, compared with the Normal group, rats in the Model group travelled significantly less distance in the open field. Compared with the Model group, all the drug intervention groups showed significant increases in total distance travelled. As shown in Figure 2B, compared with the Normal group, travel distances were significantly lower in the Model group. Compared with the Model group, the total distance travelled in the FLX group and FLX+BTVCs group were significantly increased (*P* < 0.05). The BTVCs group displayed a significantly increased total distance travelled (*P* < 0.05). As shown in Figure 2C, compared with the Normal group, the Model group displayed a significant trend toward decreased movement in the central area, but this difference was not statistical significance (*P* > 0.05). The distance travelled in the central area was significantly increased in the FLX group and FLX+BTVCs group than in the Model group (*P* < 0.05). The BTVCs group displayed a trend toward increased movement in the central area, but this difference was not statistical significance (*P* > 0.05). As shown in Figure 2D, the immobility time in the CUMS group was significantly higher than in the Normal group (*P* < 0.05). Compared with the Model group, the Normal group and drug intervention groups had significantly shorter latencies; among them, the latency of the FLX group and FLX+BTVCs group showed significant difference (*P* < 0.05), shorter than that of the BTVCs group.

The traces in the EPM test are shown in Figure 2E. Compared with the Normal group, the Model group entered significantly fewer open arms and travelled significantly lower distances in the open arms. Compared with the Model group, the drug intervention groups exhibited a higher number of entries into the open arms and travelled further in the open arms. Compared with the FLX group, the BTVCs group exhibited a denser trace (Figure 2E). The percentage of open arm entries significantly decreased in the model group than in the Normal group (*P* < 0.05); While the percentage was reversed dramatically by the FLX treatment, and the percentage in the BTVCs group of entries of the open arms further increased significantly (*P* < 0.05, Figure 2F). Compared with the Normal group, the Model group decreased the percentage of time spent in the open arms, but the behaviour of the drug intervention groups was improved by treatment. The drug intervention groups spent more time in the open arms. Compared with the Model group, the BTVCs groups spent more time exploring the open arms (P < 0.05, Figure 2G), but there were no significant differences among the drug intervention groups (*P* > 0.05, Figure 2G).

### Bifid triple viable capsules protects the hippocampus of chronic unpredictable mild stress rats

**FIGURE 3:**
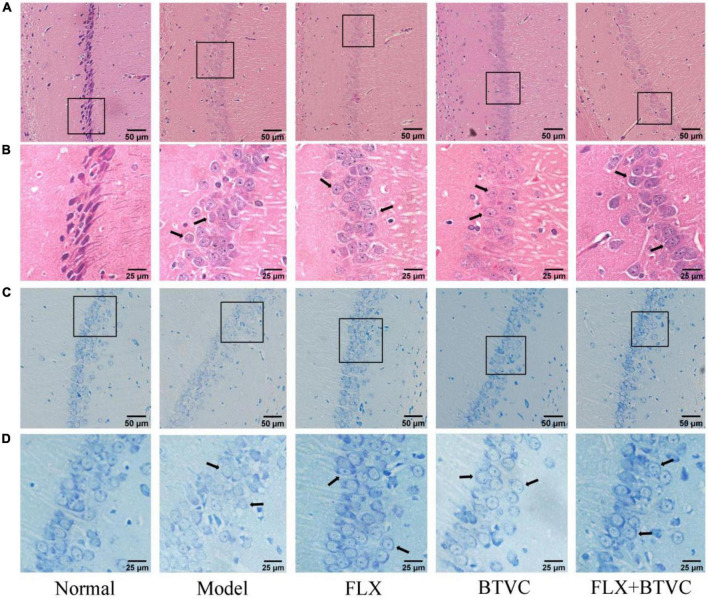
Hematoxylin and eosin staining of rat hippocampus region of each group, **(A)** 100× magnification, scale bar = 50 μm, **(B)** 200× magnification, scale bar = 25 μm; Nissl staining of rat hippocampus region of each group, **(C)** 100× magnification, scale bar = 50 μm, **(D)** 200× magnification, scale bar = 25 μm.

The results showed that the cell nucleus was full and clear, and the cells were arranged closely and neatly in the Normal group. Cells from the Model group lacked nuclei, were disordered, shrunken, and irregularly arranged, and displayed a tendency to spread to the outer layer (black arrows). We found that treatment improved the arrangement of cells compared to that of the Model group. Hippocampal cells had a normal structure, clear nucleus, regular and tighter cell arrangement, and normal cytoarchitecture, and damage to the hippocampal structure was decreased in the drug intervention groups (black arrows), shown in Figures 3A,B.

The results of Nissl staining are shown in Figures 3C,D. Compared with those of the Normal group, Model group displayed more pyramidal cells, ambiguous edges, loose and disordered cell arrangement; disappeared nuclei, reduced and diffused nissl bodies, and lighter staining (black arrows). Compared with the Model group, the hippocampal pyramidal cells of the drug treatment groups were neatly arranged, had distinct edges and nucleus, and nissl bodies were observed with the number recoveries (Figure 3D) and normal cytoarchitecture (black arrows).

### Metabolomics analysis

#### Multivariate data analysis

**FIGURE 4:**
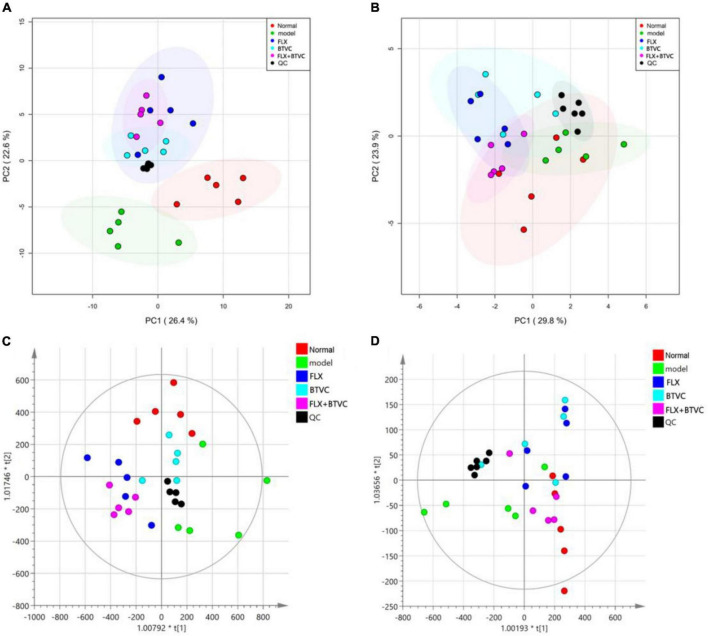
**(A)** Positive ion mode PCA score map; **(B)** negative ion mode PCA score map, **(C)** positive ion mode OPLS-DA score; **(D)** negative ion mode OPLS-DA score.

A metabolomics analysis of all samples was performed by principal component analysis (PCA) in this experiment. The PCA score plot in positive ion mode indicated that the Model group was distinct from the Normal group (Figure 4A). The negative ion mode PCA score plot is shown in Figure 4B. Although the Model group overlapped with the Normal group and the drug treatment groups, a trend toward separation was observed, indicating that the establishment of the CUMS model changed rat serum metabolites, and thus the model was successfully established. The QC sample contained an equal amount of all tested samples, as shown in Figures 4A,B. QC samples were tightly gathered, indicating that the instrument was stable and had good repeatability.

The OPLS-DA model of each group was established, and Figures 4C,D show that all experimental groups had distinct LC–MS metabolic profiles in positive and negative ion modes. This result showed that BTVC treatment was beneficial for the rat CUMS model. A permutation test was used to validate the PLS-DA model. The OPLS-DA in the positive ion mode of this experiment was *R*^2^X = 0.754, *R*^2^Y = 0.651, and *Q*^2^Y = 0.388, while the negative ion mode was *R*^2^X = 0.968, *R*^2^Y = 0.358, and *Q*^2^Y = 0.23, and the group information in the model was credible.

#### Identification of differences metabolites

Through the PCA and OPLS-DA score plots, we detected changes in serum metabolites in rats exposed to CUMS. Differential metabolites were identified by a VIP > 1.0 or VIP < 0.5, and *P* < 0.05. A total of 20 differential metabolites were detected (Table 1). Compared with the Normal group, in the Model group PC [14:0/20:1 (11Z)], lysoPC (24:0), taurohyocholate, 3-dehydro-2-deoxyecdysone, PC (O-14:0/O-1:0), glycocholic acid hydrate, Sclareol, mesterolone, oleoylethanolamide, eicosapentaenoic acid, and vetiveryl acetate showed significant changes. BVTCs regulated 6 metabolites: D-phenylalanine, methoxyeugenol, (±)-myristoylcarnitine, 18:3 (6Z, 9Z, 12Z)/P-18:1 (11Z), propionyl-L-carnitine, and arachidonic acid (AA). FLX regulated 6 metabolites: D-phenylalanine, methoxyeugenol, (±) myristoylcarnitine, PE [18:3 (6Z, 9Z, 12Z)/P-18:1 (11Z)] picotamide monohydrate 80530-63-8, and PA (13:0/22:0). FL+BTVCs regulated three metabolites: methoxyeugenol, (±)-myristoylcarnitine, and kalihinol A. Kalihinol A was only found to be a differential metabolite in the FLX+BTVCs group (Table 1).

**TABLE 1:** Serum metabolites with significant changes in treated rats.

Metabolite	Normal compared with model	FLX compared with model	BTVCs compared with model	FLX + BTVCs compared with model
PC [14:0/20:1 (11Z)]	↑*	↓	↓	↓
LysoPC (24:0)	↓*	↓	↑	↑
Taurohyocholate	↑*	↑	↑	↑
3-Dehydro-2-deoxyecdysone	↑*	↑	↑	↓
PC (O-14:0/O-1:0)	↓*	↓	↓	↓
Glycocholic acid hydrate	↓*	↓	↓	↓
Sclareol	↓*	↓	↓	↓
Mesterolone	↓*	↓	↓	↓
Oleoylethanolamide	↓*	↓	↓	↓
Eicosapentaenoic acid	↓*	↓	↓	↓
Vetiveryl acetate	↓*	↓	↓	↓
D-Phenylalanine	↑*	↑*	↑*	↑
Methoxyeugenol	↑*	↑*	↑***	↑***
(±)-Myristoylcarnitine	↓*	↓*	↓***	↓***
PE [18:3 (6Z, 9Z, 12Z)/P-18:1 (11Z)]	↑*	↑**	↑**	↑
Propionyl-L-carnitine	↑*	↑	↑*	↑
Arachidonic acid	↓*	↓	↓*	↓
Picotamide monohydrate	↑*	↑**	↑	↑
PA (13:0/22:0)	↓*	↓*	↓	↓
Kalihinol A	↓*	↓	↓	↓*

Compared with the model group, “↑,” the expression level is up-regulated; “↓,” the expression level is down-regulated.

*P < 0.05, **P < 0.01, and ***P < 0.001.

#### Metabolic pathway analysis

**FIGURE 5:**
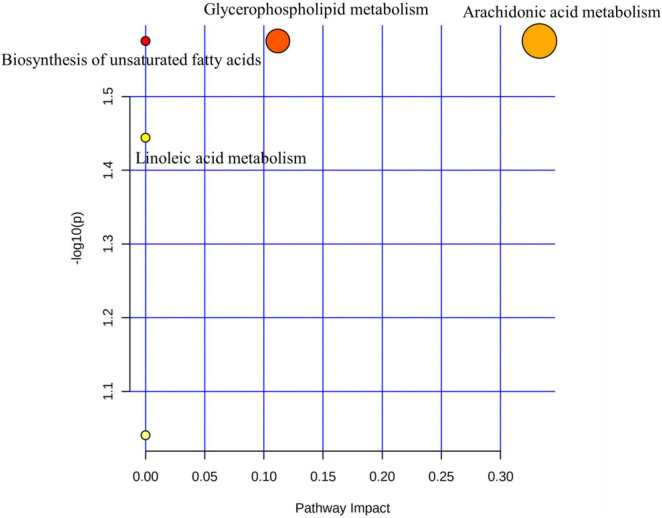
Metabolic pathway analysis.

The 20 KEGG-annotated differential metabolites were imported into MetaboAnalyst to analyse the metabolic pathway.^2^ The pathway analysis is summarised in Figure 5, showing the biosynthesis of unsaturated fatty acids, glycerophospholipid metabolism, linoleic acid metabolism, and AA metabolism. Biosynthesis of unsaturated fatty acids and AA metabolism pathways could be mediated by BTVC.

#### The network function analysis by ingenuity pathway analysis

**FIGURE 6:**
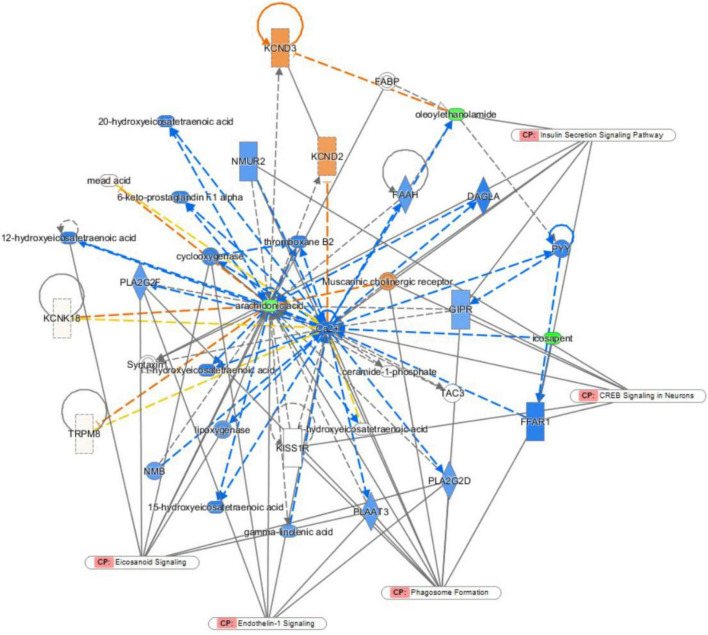
Ingenuity pathway analysis (IPA) analysis.

Ingenuity pathway analysis was applied to discover potential biomarkers of depression. In the network function analysis, the related metabolites tended to gather into a single network (Figure 6). The pathways were cyclic adenosine monophosphate response element binding protein (CREB) signalling, phagosome formation, endothelin-1 (ET-1) signalling, eicosanoid signalling, and insulin secretion signalling pathways in neurons. The results indicate that these altered pathways may be involved in depression development.

## Discussion

As previously reported by others, CUMS is currently a well-recognised animal model of depression, and the animal model established can stably simulate the symptom of human depression (23). In the present study, the CUMS depression model was constructed by stimulating animals with chronic random stressors to induce a depressive state, leading to prolonged immobility and reduced exploration. The drug intervention ameliorated these effects; the distance travelled and exploration increased. HE and Nissl staining were used to observe the morphology of hippocampal tissue in each group and evaluate the therapeutic effect of the intervention. A grate number of neurons in CUMS rats were damaged, and Nissl-positive cells were remarkably reduced in the hippocampi. Compared with the Model group, we found that the severity of hippocampal pyramidal cell and nissl body damage induced by CUMS was ameliorated by BTVCs treatment. All of the results above indicate an antidepressant effect of BTVCs. Recent studies demonstrated that psychobiotics or prebiotics may be more specific and beneficial to depression. In a placebo-controlled trial, Pinto-Sanchez et al. discovered that the probiotic *Bifidobacterium longum* reduced depression scores and increased the quality of life in patients with Irritable Bowel Syndrome (26). However, this probiotic is now not available on the market. In another randomised clinical trial, Bifidobacterium *breve* CCFM1025 showed an antidepressant effect in patients with major depression disorder (27, 28). Similarly, Yang et al. demonstrated that supplementation of Bifidobacterium reversed depression-like behaviours, social interaction and sucrose preference, in mice with chronic social defeat stress (29). In this regard, intake of Bifidobacterium may prevent the onset of depression and relapse in depressed patients and animals.

Changes in serum metabolites were identified by Untargeted Metabolomics techniques based on LC–MS to determine the potential mechanism of BTVCs in the treatment of depression. We identified 20 depression-related biomarkers by serum metabolomics, six of which were significantly regulated by BTVC. The serum-differentiated metabolites were enriched in four metabolic pathways that include the glycerophospholipid metabolism, AA metabolism, linoleic acid metabolism, and biosynthesis of unsaturated fatty acids.

Glycerophospholipid metabolism and AA metabolism are the most profound pathways (*p* < 0.05, impact >0.1) in the pathway analysis. Glycerophospholipids are important constituents of the brain and regulate brain function. Zheng et al. demonstrated that glycerophospholipid might participate in the onset of depression-like behaviours in female cynomolgus macaques (30). Tian et al. identified glycerophospholipid metabolism as a vital cause of depression-like behaviours in mice (31). Additionally, Zhang et al. elucidated that dysfunctions of glycerophospholipid and the related metabolic enzymes may account for the depression in CUMS rat model (32). Consistent with the literature, this research found that PE [18:3 (6Z, 9Z, 12Z)/P-18:1 (11Z)] descended in the model group compared to the Normal group, but was reversed after BTVC intervention.

PE [18:3 (6Z, 9Z, 12Z)/P-18:1 (11Z)], in particular, consists of a γ-linolenic acid chain at the C-1 position, a plasmalogen 18: 1n7 scaffold at the C-2 position, and a phosphorylethanolamine moiety at the C-3 position. γ-linolenic acid that differs from the saturated fatty acid on C-1 of most phospholipids belongs to an omega-6 fatty acid. Its elongation product in the human body, Dihomo-γ-linolenic acid, may contribute to a lower risk of depression (33). Phosphatidylethanolamine, a major constituent of cell membranes in the brain, is associated with depression (34) and chronic stress (35).

Glycerophospholipid can be catalysed by phospholipase A2 to produce AA (36). AA metabolism has also been associated with depression severity (37, 38). AA is one of the most abundant polyunsaturated fatty acids (PUFAs) in vertebrates, and can affect depression severity through modulating 5-HTT binding potential (37). AA is metabolised to potent signalling molecules, including leukotrienes and prostaglandins, that mediate responses to physiological stresses, such as inflammation, and play an important role in the immune inflammation mechanism of depression (39, 40). In our study, the concentration of AA in the serum samples of the Model group and intervention groups was higher than that of the Normal group, and the inflammatory reaction of the Model group was more intense than that of the other groups.

Among the serum-differentiated metabolites, phenylalanine is a precursor of catecholamine that can act as a neurotransmitter and an epinephrine-like substance and plays a pivotal role in depression (41). D-phenylalanine administration has been reported to rapidly activate extracellular signal-regulated kinase (ERK) pathways, a critical step for memory formation, in the cortex and hippocampus, two brain areas involved in memory processing (42). In our study, the serum D-phenylalanine concentration in the Model group decreased after CUMS and was significantly improved after probiotic intervention, indicating that BTVCs may improve depressive symptoms by modulating D-phenylalanine metabolism.

L-carnitine is an endogenous substance that acts as a carrier for fatty acids across the inner mitochondrial membrane, and propionyl-L-carnitine (PLC) is an ester of L-carnitine required for the transport of fatty acids into the mitochondria (43). PLC is a member of the most abundant group of carnitines in the body, comprising more than 50% of all acylcarnitines quantified in tissues and biofluids (44). PLC attenuates forebrain ischaemia-induced neuronal injury, oxidative stress, and energy depletion in the hippocampal CA1 region (45). Changes in acylcarnitine metabolites represent the metabolic status in the brain (46). The biological function of myristoylcarnitine is unclear, yet studies showed that myristoylcarnitine significantly increased when the hippocampus was damaged (47). A significantly increased concentration of myristoylcarnitine in the serum of Model group rats was observed in this experiment, which is consistent with earlier findings.

To further explore the mechanism of BTVCs, this study used IPA to establish a biomolecular interaction network. CREB signalling in neurons, phagosome formation, endothelin-1 signalling, eicosanoid signalling and insulin secretion signalling pathways in other cells were highlighted by the IPA. ET-1 has been reported to evoke necrotic neuronal damage and cause reactive nitrogen species-mediated tissue injury (48). ET-1 in CA1 was critical for regulating the excitability of CA1 pyramidal neurons. Upregulation of ET-1 expression reduced the excitability of CA1 pyramidal neurons and decreased excitatory neurotransmission (49). CREB is a nuclear regulatory factor in eukaryotic cells. The impaired CREB signalling pathway is associated with the progression of depression (50). The activity and expression of CREB in brain tissues are markedly reduced, and its restoration may be responsible for the therapeutic effect of antidepressants (51). In the hippocampus, CREB is crucial mediator of antidepressant effects. A wide variety of standard antidepressant treatments increase CREB activity within the hippocampus, and accumulating evidence suggests roles for CREB-regulated expression of neural growth factors (52).

Bifid triple viable capsules may be a potential adjuvant therapy for depression. This study provides a new insight into the development of antidepressants with fewer side effects than traditional treatments, as it revealed the interconnected metabolic influence of BTVCs in CUMS rats and provided vital evidence for the antidepressant efficacy of BTVCs *via* the Biosynthesis of unsaturated fatty acids and AA metabolism pathways. CREB signalling in neurons and endothelin-1 signalling in neurons may be involved in the development of depression. In addition, further studies that examine the therapeutic effect of BTVCs in humans may be merited. According to the results of IPA, further molecular biology experiments should be carried out.

## Conclusion

The present study demonstrated that BTVCs can significantly alleviate depression-like behaviours and decrease hippocampal structural damage. In addition, our results elucidated that BTVCs modulated glycerophospholipid metabolism, linoleic acid metabolism, AA metabolism, and biosynthesis of unsaturated fatty acids in CUMS rats. Furthermore, IPA analysis showed that BVTCs alleviate depression by regulating endothelial-1 signalling and CREB signalling pathways. This study has provided a deeper understanding of microbiota-related metabolic processes in the treatment and prevention of depression and has, at least partially, elucidated the anti-depressant mechanism of BTVCs.

## Data availability statement

The original contributions presented in this study are included in the article/Supplementary material, further inquiries can be directed to the corresponding authors.

## Ethics statement

The animal study was reviewed and approved by the Ethical Committee in Research Medical College of China Three Gorges University of Medical Sciences.

## Author contributions

QB and JZ: investigation, formal analysis, data curation, and writing—original draft. XG: software and visualisation. YF and HY: writing—original draft. WC: resources. ZF: conceptualisation, supervision, and writing—review. MC: conceptualisation, supervision, writing—review, and funding acquisition. All authors contributed to the article and approved the submitted version.

## Footnotes

1
http://www.ingenuity.com

2
https://www.metaboanalyst.ca/
