# Supplementary material for: The antidepressant effects and serum metabonomics of bifid triple viable capsule in a rat model of chronic unpredictable mild stress
Source: Front Nutr. 2022 Sep 15;9:947697. doi: 10.3389/fnut.2022.947697 (PMC9520780; doi:10.3389/fnut.2022.947697)
Supplement: Supplementary file 1 [file Table_1.DOCX]

**1. Sample Preparation**

Operate on ice, accurately pipette 50 μL of serum sample into a 1.5 mL EP tube, and record its number and sampling amount. Add 200μL of cold methanol (including internal standard), vortex for 2 min, and let stand for 10min at low temperature. Centrifuge at 14000 g for 15 min at 4 ℃. Aspirate 200 μL of the supernatant and place it in a new EP tube. After the sample is concentrated by low-temperature centrifugation, it is stored in a refrigerator at - 20 °C for later use. Before performing computer analysis, reconstitute the concentrated extract sample with 100 μL of 20% methanol/water solution until it is completely dissolved, then centrifuge to collect the supernatant, and perform positive and negative ion mode analysis.

**2. Analysis Conditions of LC/MS**

Positive ion mode: Chromatographic column: BEH C8 column (1.7μm, 2.1×100mm) American Waters Company; column temperature: 50℃; injection volume: 5μL; flow rate: 0.35 mL/min; mobile phase A: 0.1% formic acid/ Water; mobile phase B phase: 0.1% formic acid/acetonitrile; gradient elution program: 5% phase B is the initial concentration, 0–1 min, phase B stays at 5%, 1.1–11 min, phase B changes from 5% 100%, 11.1-13min, phase B stays at 100%, 13.1-15min, phase B stays at 5%.

Negative ion mode: Chromatographic column: HSS T3 column (1.8μm, 2.1×100 mm) American Waters Company; column temperature: 50 ℃; injection volume: 5 μL; flow rate: 0.35 mL/min; mobile phase A: 0.1% formic acid/water ; Mobile phase B phase: 0.1% formic acid/acetonitrile; Gradient elution program: 5% phase B is the starting concentration, 0–1 min, phase B stays at 5%, 1.1–11min, phase B changes from 5% to 100 %, 11.1-13 min, phase B stays at 100%, 13.1-15min, phase B stays at 5%.

Mass spectrometry conditions: first-level full scan of mass spectrometry + IDA second-order product ion scan-positive ion mode: ESI-Positive mode of electrospray ion source, first-level full scan + IDA second-order product ion scan mode. Spray Voltage (V): 5000; Temperature (℃): 650; CUR: 30.000; GS1: 60.000; GS2: 60.000; Mass range(m/z): TOF-MS(100-1200); TOF-MS2(50- 1200); TopN: 12.

Mass spectrometry primary full scan + IDA secondary product ion scan-negative ion mode: ESI-Negative mode of electrospray ion source is adopted, primary full scan + IDA secondary product ion scan mode is adopted. Spray Voltage (V): -4500; Temperature (℃): 650; CUR: 30.000; GS1: 60.000; GS2: 60.000; Mass range (m/z): TOF-MS(100-1200); TOF-MS2(50 -1200); TopN: 12.

**3.Data Collection**

All measurement data are collected by Analyst® TF data acquisition software (AB SCIEX Corporation, USA).

**4.Data Processing**

The first-level mzmL file and the second-level mgf file, the peak table and peak detection are extracted with OneMap-PTO software parameters, and the second-level uses precipitation to retain 50. Features include standard datebase (OSI-SMMS), KEGG library and network extension datebase (integrated HMDB/Metlin/GNPS/lipidblast/Massbank/MMCD and other network datebase resource integration). Import the multivariate data into the SIMCA software package to perform the Orthogonal Least Squares Method (OPLS-DA) discriminant model bias analysis to generate the variable information that contributes to the transformation assignment classification and the transformation navigation classification. After testing, use the R^2^X (variables in which the latent variable in the model reflects the independent variable X (variable), the variable of the R^2^Y latent variable) and Q2 (the predictability of the latent variable in the model) to make an effective variable case for the model, these 3 parameter values ​​are fighting, and R^2^Y/Q^2^ is close to 1, indicating that this model is reliable. Variable important in the projection (VIP) to determine the contribution of each predicted data to the model, VIP>1, and t-test is used at the same time to screen the components with *P*<0.05, which means it has a sensitive meaning. Can be used as a potential biomarker. Eventually, biomarkers with special significance can be captured. The chemical composition database of the substance, collected by Software Analyst TF 1.6, and then filtered by the XIC Manager function of PeakViewTM software to retain time (effect range within 45 seconds) and mass-to-charge ratio (m/z) (performance range within 10 ppm). Determined compound. Combining the information of the first-level particle and the second-level particle of the compound in a certain process, and the matching rate (80%), determine the attribution of a single particle, thereby completing the final judgment of the object. Finally, the possible universe changes are analyzed in the MetaboAnalyst database.
